# Multilevel resistive switching memory in lead-free double perovskite La$_{2}$NiFeO$_{6}$ films

**DOI:** 10.1186/s11671-023-03885-7

**Published:** 2023-08-29

**Authors:** Yongfu Qin, Yuan Gao, Fengzhen Lv, Fangfang Huang, Fuchi Liu, Tingting Zhong, Yuhang Cui, Xuedong Tian

**Affiliations:** College of Physical Science and Technology and Guangxi Key Laboratory of Nuclear Physics and Technology, Guangxi Normal University, Yucai Road, Guilin, 541000 China

**Keywords:** La$_{2}$NiFeO$_{6}$, Multilevel resistive switching, Oxygen vacancy, Space-charge-limited current, Schottky-like barrier

## Abstract

Dense and flat La$_{2}$NiFeO$_{6}$ (LNFO) films were fabricated on the indium tin oxide-coated glass (ITO/glass) substrate by sol–gel method. The bipolar resistive switching behavior (BRS) could be maintained in 100 cycles and remained after 30 days, indicating that the LNFO-based RS device owned good memory stability. Surprisingly, the multilevel RS characteristics were firstly observed in the Au/LNFO/ITO/glass device. The high resistance states (HRSs) and low resistance state (LRS) with the maximum ratio of $\sim$ 500 could be remained stably in 900 s and 130 cycles, demonstrating the fine retention and endurance ability of this LNFO-based RS device. The BRS behavior of Au/LNFO/ITO/glass devices primarily obeyed the SCLC mechanism controlled by oxygen vacancies (OVs) dispersed in the LNFO layer. Under the external electric field, injected electrons were captured or discharged by OVs during trapping or detrapping process in the LNFO layer. Thus, the resistive state switched between HRS and LRS reversibly. Moreover, the modulation of Schottky-like barrier formed at the Au/LNFO interface was contributed to the resistive states switchover. It was related to the change in OVs located at the dissipative region near the Au/LNFO interface. The multilevel RS ability of LNFO-based devices in this work provides an opportunity for researching deeply on the high density RS memory in lead-free double perovskite oxides-based devices.

**Supplementary Information:**

The online version contains supplementary material available at 10.1186/s11671-023-03885-7.

## Introduction

With the popularity of portable mobile electronic communication, the updating and upgrading of communication devices are more reliant on high density, fast speed and low consumption memories. Among various potential memory technologies, resistance random access memory (RRAM), comprising a semiconductor/insulator sandwich structure with electrodes, is considered as an ideal candidate due to its low size-dependence, high memory-density and device-integration [1–3]. The basic feature of RRAM is the ability to switch different resistance states of the device simply by applying a voltage or current pulse. Lots of materials have been reported that possess resistive switching (RS) characteristics [4–6]. Therein, transition metal oxides with the perovskite structure (ABO$_{3}$, A = rare-earth or alkaline-earth metal cation, B = transition metal cation) occupy an important position [7–9]. In ABO$_{3}$, the competing phenomena between various electronic phases triggers the dramatic change of resistance under external electric field, offering an opportunity for the high-density data storage by RS memory mode [9–11].

In order to further explore and develop the properties of ABO$_{3}$ in electronics, partial substitution of ions at B-site is an effect way [12]. Thus, double-perovskite transition-metal oxides, A$_{2}$B$^{\prime }$B$^{\prime \prime }$O$_{6}$, as derivatives of ABO$_{3}$ are attracted more and more attention recently. On the one hand, because of the flexibility of numerous possible substitutions at B$^{\prime }$ and/or B$^{\prime \prime }$ sites, some novel physical phenomena and characteristics can be observed in A$_{2}$B$^{\prime }$B$^{\prime \prime }$O$_{6}$, such as colossal magneto-resistance, magneto-capacitance, multiferroic and so on [12–14]. On the other hand, the B$^{\prime }$O$_{6}$ or B$^{\prime \prime }$O$_{6}$ octahedron can be expanded/contracted and tilted, and then, many vacancies will be generated inside the crystal, which is beneficial to stimulate interesting electric conductive behavior in the A$_{2}$B$^{\prime }$B$^{\prime \prime }$O$_{6}$ layer [15–17]. Since H. L. Zhou et al. reported the bidirectional adjustable multistage RS characteristics and photo-induced negative differential resistance effect in Bi$_{2}$FeCrO$_{6}$ films, the potential of RS memory in double-perovksite oxides has been attracted attention gradually [18, 19]. However, limited reports about memory field are not helpful for further researching and developing the RS memory function of double-perovksite oxides.

In this work, we researched the RS characteristics on La$_{2}$NiFeO$_{6}$ (LNFO), which is a young member in double-perovskite oxides. Although the electrochemical and ferromagnetic properties of LNFO have been reported, [12, 20] RS memory ability has not been researched. In our work, LNFO films with high density and coverage were prepared by sol–gel method on indium tin oxide-coated galss substrates (ITO/glass). Under bias voltages sweeping, the typical bipolar RS (BRS) behavior was observed in the LNFO film. And the BRS behavior of Au/LNFO/ITO/glass devices could be remained in 100 cycles and 30 days at room temperature in the air. Surprisingly, four resistance states, one low resistance state (LRS) and three high resistance states (HRSs), were firstly observed under different reset voltages ($V_\textrm{resets}$). The maximum ratio of HRSs and LRS was $\sim$500. And this multilevel RS effect could be maintained stably over 900 s and 130 cycles. According to analyzing, the RS behavior was primarily attributed to the trap-controlled space-charge-limited current (SCLC) mechanism caused by oxygen vacancies (OVs) in the LNFO medium layer. And the change in Schottky-like barrier between Au electrode and LNFO layer was contributed to the resistance states switching. This work can help to develop members of double perovskite oxides researched in RS memory, and provide an opportunity to develop the high-density storage ability in double perovskites-based memory devices.

## Methods and characterization

### Exprerimental-sample preparation

LNFO films were synthesized by sol–gel method using [La(NO$_{3}$)$_{3}\cdot$6 H$_{2}$O], [Ni(NO$_{3}$)$_{2}\cdot$6 H$_{2}$O] and [Fe(NO$_{3}$)$_{3}\cdot$9 H$_{2}$O] with molar ratio of 2:1:1. Above solutes were individually dissolved in 2-methoxyethanol (2.5 ml). Subsequently, above solution was stirred for $\sim$3 h under ambient condition, The final precursor-solution was gained after quietly placing 6 h. The ITO/glass conductive glass with 10$\times$10 mm$^{2}$ area was adopted as the fabricated substrate of the LNFO film. ITO/glass conductive substrate was cleaned by sequential deionized water, ethanol and sonication in acetone. After drying in air, the precursor gel was spin-coated on the cleaned substrate at 4000 rpm for 50 s. And then, the above sample was heated at 350 $^{\circ }$C for 30 min. Finally, the target LNFO film was obtained with the stable crystal structure after annealing at 600 $^{\circ }$C for 3 h in the muffle furnace.

### Characterization

The crystalline structure of LNFO samples was identified using a Rigaku Laboratory X-ray diffractometry (XRD)-MiniFlex600 instrument. The source of radiation is Cu K$\alpha _{1}$. The chemical valence state analysis of LNFO films was performed using X-ray photoelectron spectroscopy with Al K$\alpha$ radiation (XPS; ESCALAB250Xi, Thermo Fisher Scientific, USA). The energy band structure of LNFO samples was investigated by ultraviolet photoelectron spectroscopy (UPS) measurement, performed with the ESCALAB250Xi spectrometer of Thermo Fisher Scientific, USA. UPS measurement was performed using He I (21.22 eV) under a negative bias voltage ($-$ 5 V), and the spectra were calibrated against the silver Fermi level. The morphology of LNFO films was investigated by scanning electron microscopy (SEM; Quanta200, FEI, USA) and atomic force microscopy (AFM; Multimode 8, Bruker, GER). The RS performance of LNFO-based devices were monitored by an electrical measurement system, carrying the Keithley 2410 SourceMeter and a micromanipulated cryogenic probe system (ST-500-4TX-6PORTS, Janis Research, USA).

## Results and discussion

**Fig. 1:**
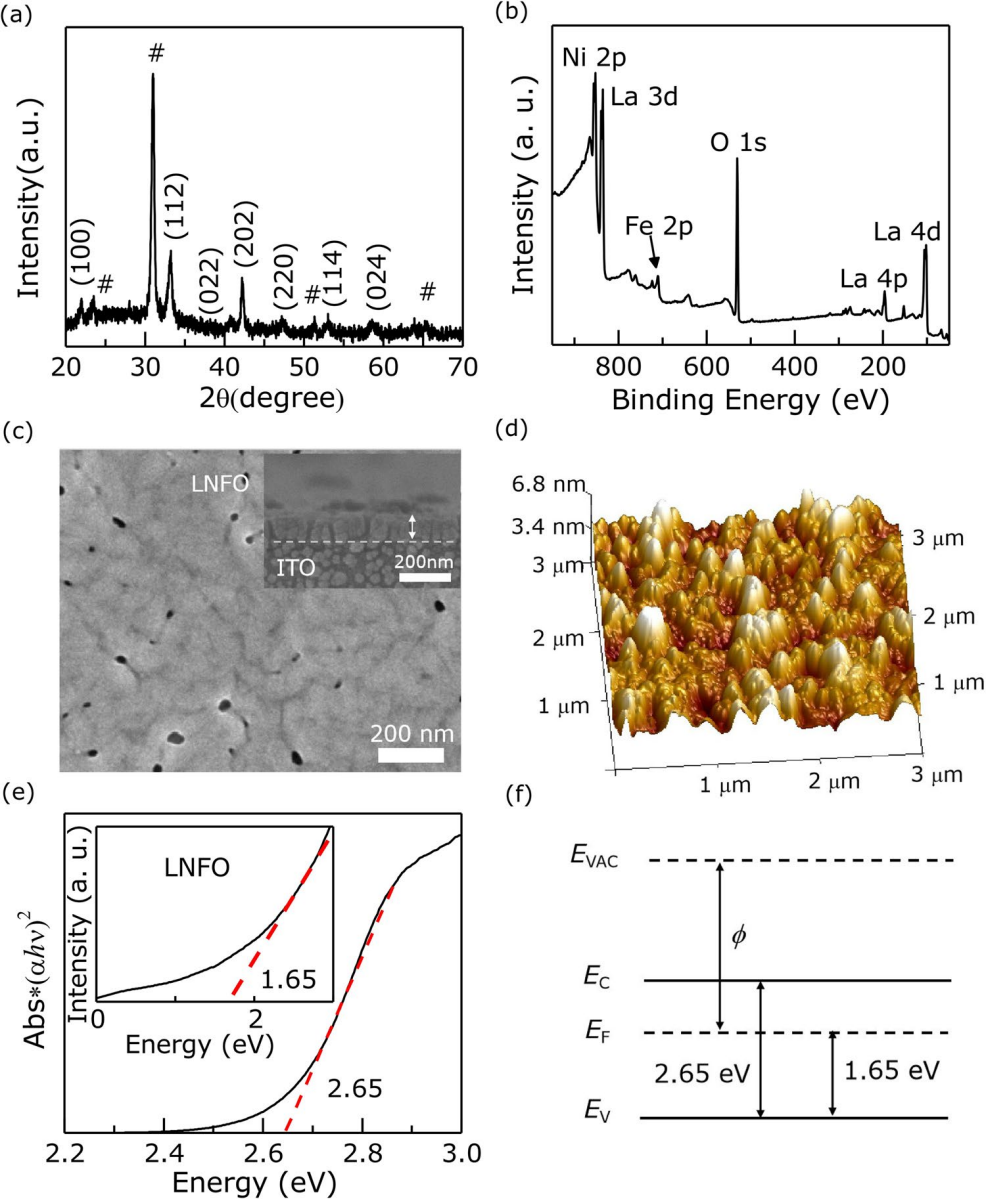
**a** Room-temperature XRD patterns of LNFO/ITO heterojunction. $\sharp$ represents the peak of ITO substrate. **b** The XPS wide spectrum of LNFO film. **c** The SEM surface image of LNFO sample and the inset is the cross-sectional SEM micrograph of LNFO/ITO heterojunction. **d** Three-dimensional AFM rendering of the topographic image of LNFO. **e** Tauc plot obtained from UV–Vis spectroscopy. The inset shows the UPS spectrum in the VB region. **f** The schematic diagram of band structure in LNFO

The crystalline structure of the LNFO samples was analyzed by XRD patterns. As shown in Fig. 1a, the XRD pattern of LNFO samples is well indexed into the monoclinic structure of LNFO with the space group $\textit{P}$2$_{1}$/$\textit{n}$ [12, 21]. And the estimated values of lattice parameters are a = 5.8554 Å, b = 5.0904 Å, c = 7.9348 Å, $\alpha$ = $\gamma$ = 90$^{\circ }$, $\beta$ = 95.24$^{\circ }$. Figure 1b shows the wide XPS spectrum of LNFO film. Obviously, the spectrum only presents La, Ni, Fe, and O elements, indicating that LNFO films are pure chemically. The SEM image taken on the top of as-grown samples clearly presents that the LNFO film with full uniformity covered the substrate in Fig. 1c. And the across-sectional of LNFO/ITO heterojunction shows that the thickness of LNFO is about 120 nm in the inset of Fig. 1c. In order to further investigate the quality of LNFO films, AFM was carried out on demonstrating the roughness and uniformity of samples. As illustrated in Fig. 1d, the three-dimensional topographic image of LNFO films exhibits that the root-mean-square roughness is $\sim$3.42 nm in a typical scanning area of 3 $\mu$m$\times$3 $\mu$m. It further indicates that LNFO samples possess the smooth surface and uniform grains. Figure 1e presents the Tauc plot obtained from the UV–Vis spectrum of LNFO film. The band gap is determined to 2.65 eV and the valence band maxima is 1.65 eV, which is close to the theoretical value [20]. The band structure of LNFO presents that the Fermi level of LNFO is located near the conduction band (Fig. 1f), demonstrating that LNFO is similar to *n*-type semiconductor and abundant trap states can be distributed above the valence band.

**Fig. 2:**
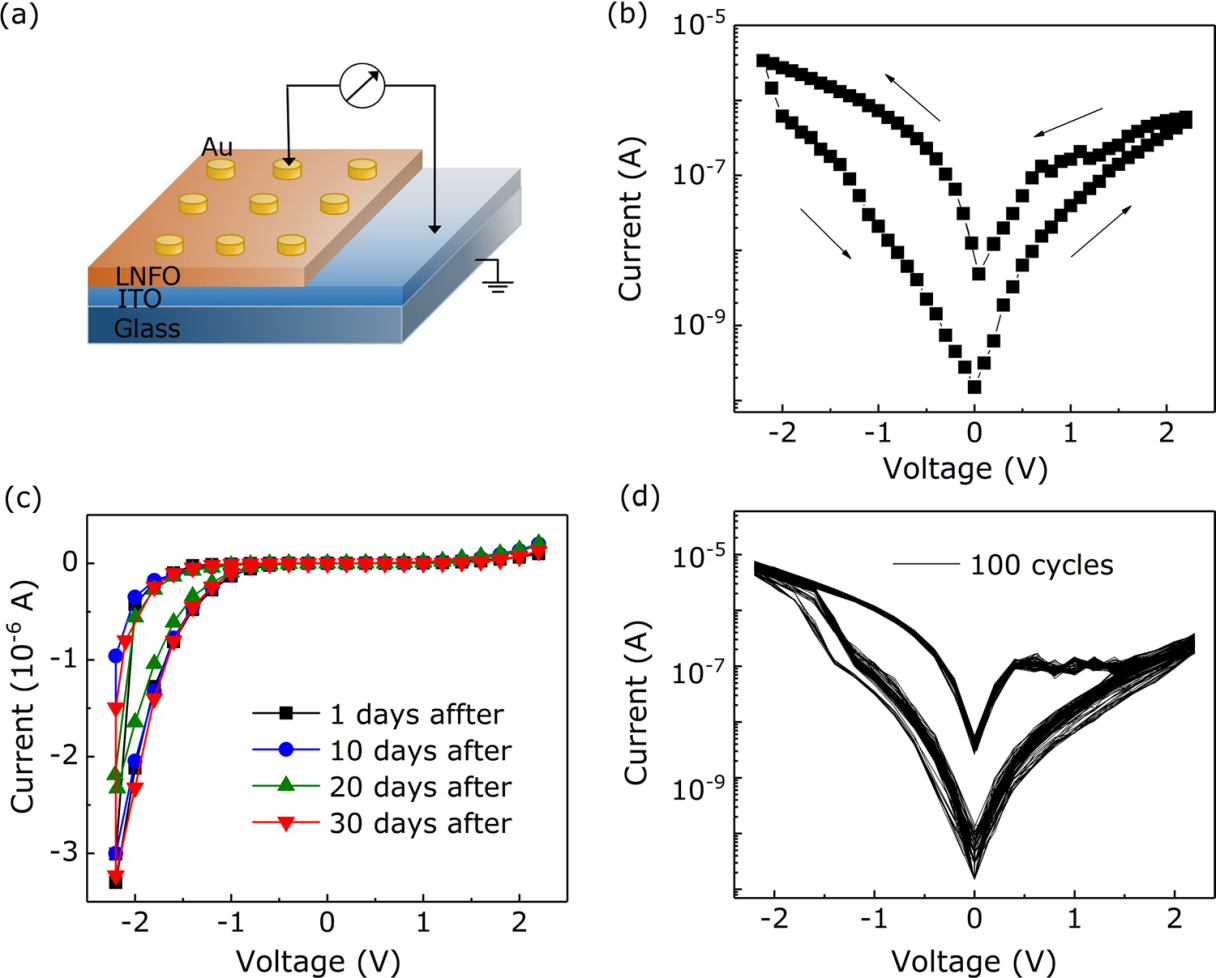
**a** The schematic device structure of Au/LNFO/ITO/glass cell for the measurement. **b** The *I–V* characteristic of Au/LNFO/ITO/glass devices under the sweeping voltage +2.2 V to $-$2.2 V. The arrows represent the direction of the sweeping voltage. **c** *I–V* curves measured in the range from 0 to 30 days at room temperature. **d** *I–V* curves of Au/LNFO/ITO/glass cells measured during 100 sweep cycles

Figure 2a presents the schematic structure of the LNFO-based device for the measurement of RS effect. The Au layer of $\sim$100 nm thickness was sputtered as the top electrode on LNFO/ITO/glass stacks. The diameter of Au electrodes was 300 $\mu$m. The voltage was controlled by one of the Au electrode under *dc* sweeping voltage applied as 0$\rightarrow$2.2 V$\rightarrow$0$\rightarrow$ $-$2.2 V$\rightarrow$0; the ITO electrode was grounded. As shown in Fig. 2b, obvious hysteresis phenomenon appears under the bias voltage sweeping. The ‘SET’ process occurs at 2.2 V, the resistance switches from HRS to LRS; the ‘RESET’ process occurs at $-$2.2 V, the resistance switches from LRS to HRS. Figure 2b presents that an obvious asymmetric *I–V* hysteresis loop between positive and negative voltage regions. It implies the formation of a Schottky-like barrier at the Au/LNFO/ITO interface [22–24]. Above *I–V* hysteresis obtained under stressing forward and reversed voltages sweep demonstrates that the LNFO-based device possesses the electronic RS memory ability. After further exposure time up to 30 days in the air (Fig. 2c), the hysteresis behavior is steady, demonstrating that the memory effect exists stably over a long time in the Au/LNFO/ITO/glass devices. As exhibited in Fig. 2d, the cycle-to-cycle variability of currents is consistent during 100 times sweeps. The different current steps of the *I–V* curves, which was observed in the positive bias sweeping region, were mainly due to the rupture of different conductive channels in the LNFO film [25]. Above results display that the Au/LNFO/ITO/glass device has the fine RS stability.

**Fig. 3:**
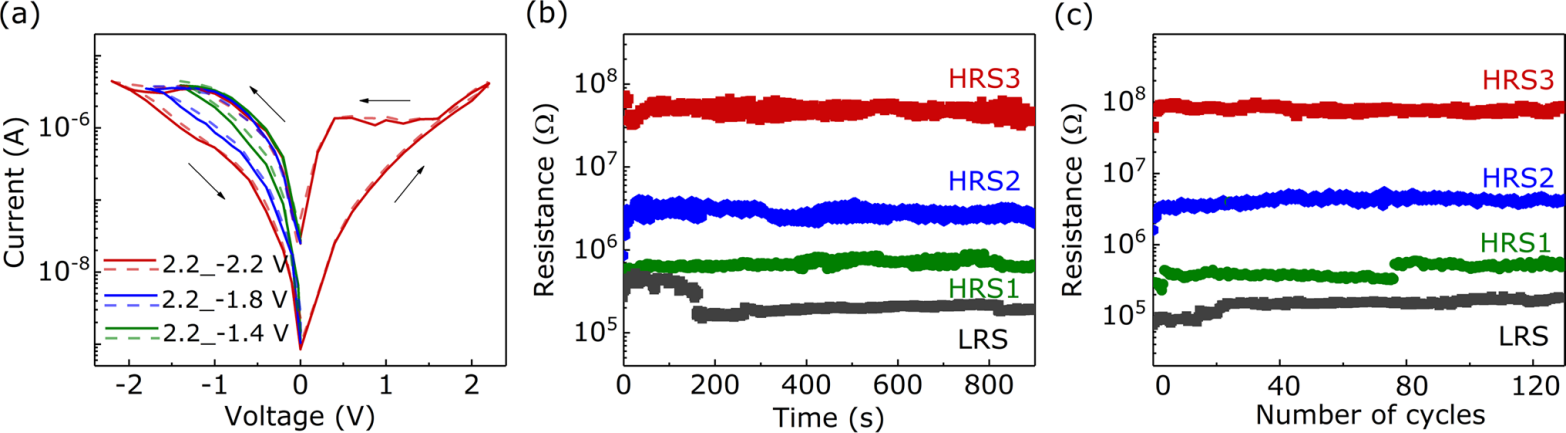
**a*** I–V* loops swept under different periodic voltages (0$\rightarrow$2.2 V$\rightarrow$ $-$1.4 V/$-$1.8 V/$-$2.2 V$\rightarrow$0) at room temperature. The solid and dashed lines represent the 1st and 60th loops, respectively. The arrows represent the direction of sweeping. **b** Resistance versus retention time measured at $-$0.2 V after poling by +2.2 V (gray line) and $-$1.4 V (green line)/$-$1.8 V (blue line)/$-$2.2 V (red line). **c** Resistance *vs*. cycles measured at $-$0.2 V under above different operating voltages

Subsequently, by applying different periodic voltages (0$\rightarrow$2.2 V$\rightarrow$ $-$1.4 V /$-$1.8 V /$-$2.2 V$\rightarrow$0) to the Au/LNFO/ITO/glass cell, four resistance states (LRS, HRS1, HRS2 and HRS3) are observed in the negative voltage region, as shown in Fig. 3a. And *I–V* loops measured in the first cycle (solid loops) and the sixtieth cycle (dashed loops) demonstrate uniformity, indicating the fine stability of multilevel RS performance in Au/LNFO/ITO/glass cells. This multilevel RS effect is observed in the LNFO-based electronic device for the first time, indicating that LNFO-based devices possess the potential of high density memory. It is well known that the retention and endurance ability are important to judge memory ability of RS devices [26, 27]. As illustrated in Fig. 3b, the data retention is evaluated with a $V_\textrm{r}$ ($-$0.2 V) under ambient condition. After the fluctuation of current for serveral minutes, LRS, HRS1, HRS2 and HRS3 recorded at the $V_\textrm{r}$ can be distinguished clearly. and these four resistance states can maintain stably in 900 s. The current fluctuation within the first minutes of measurement can be ascribed to the presence of defects in the LNFO layer. Under the influence of the external electrical field, defects tend to accumulate near the conductive channels. This phenomenon can change the shape and size of the conductive channels, thereby affecting the charge transport process and leading to the observed current fluctuation [28, 29]. The maximum ratio of HRSs and LRS is $\sim$500. To further confirm the endurance of multilevel switching in the Au/LNFO/ITO/glass device, we measured the number of cycles between different resistance states read at the $V_\textrm{r}$ under different operating voltages, 2.2 V, $-$1.4 V, $-$1.8 V and $-$2.2 V. As shown in Fig. 3c, a stable LRS and three HRSs can be distinguished and remained in 130 cycles. Above results demonstrate that the Au/LNFO/ITO/glass device possesses the potential of high stability and density of RS memory. In addition, as previous reports researching, RS characteristics were vary with different metal electrodes [30, 31]. Thus, LNFO-based memory devices using different metal top-electrodes (Ag, Pt) were fabricated. In the supporting information, the similar BRS behaviors were obtained as Au top-electrode under a sweep (0 V$\rightarrow$2.2 V$\rightarrow$ $-$2.2 V$\rightarrow$0 V) in the Ag/LNFO/ITO/glass and Pt/LNFO/ITO/glass devices, as shown in Fig. S1(a) and (b). Figure S1(c) presents the retention of Ag/LNFO/ITO/glass device measured at a low reading voltage ($V_\textrm{r}$ = $-$0.1 V) under a pulse of $-$2.2 V and 2.2 V. The ratio of HRS/LRS is $\sim$15 in 700 s, and the ratio reduces to $\sim$3.7 over 720 s. The retention of the Pt/LNFO/ITO/glass device was measured at a $V_\textrm{r}$ (0.2 V) under a pulse of $-$2.2 V and 2.2 V [Fig. S1(d)]. The ratio of HRS/LRS is just $\sim$2. Above results indicate that the RS ability in the LNFO-based devices with Ag and Pt electrodes is not better than that in the Au/LNFO/ITO/glass device.

**Fig. 4:**
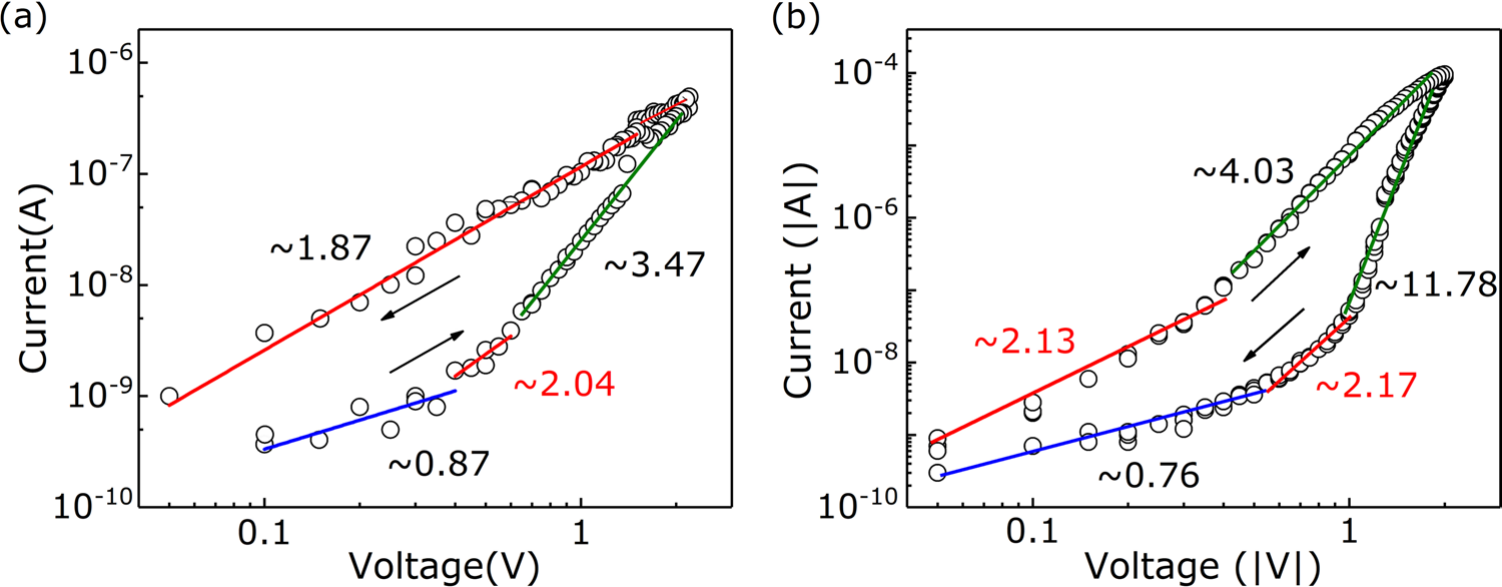
Fitted lines of the double logarithmic *I–V* plots. The arrows represent sweeping directions

Until now, the origin of multilevel RS effect is still controversial. In the previous studies, the different size of conductive filaments controlled by the maximum voltage or compliance current could induce multilevel RS property [32, 33]. We surmised that the origin of multilevel RS property in the Au/LNFO/ITO/glass device was primarily related to the electrons trapping and detrapping process under different electric field utilizing operated voltage. It suggests that the RS behavior in the Au/LNFO/ITO/glass device obeys SCLC conductive mechanism [30, 34]. In order to verify above surmise, the *I–V* curve was repoltted on a log-log scale to investigate the RS mechanism in the Au/LNFO/ITO/glass device. As illustrated in Fig. 4a, the low positive bias region (vary from 0 to 0.3 V) shows Ohmic behavior as the fitting slope ($\sim$0.87) of log(*I*) versus log(*V*) is close to 1. As the positive voltage increasing (0.3$\sim$2 V), the slopes of log(*I*)-log(*V*) curve increase to 2.04 and 3.47, respectively. This behavior obeys the trap-controlled SCLC model ($I \propto V^{2}$ and $I \propto V^{n}$) [35, 36]. When the bias voltage arrives at the set voltage ($V_\textrm{set}$ = 2.2 V), the Au/LNFO/ITO/glass device switches from HRS to LRS. The device can remain LRS even though the bias voltage sweeps reversely, as shown in Fig. 4b. When the sweeping voltage arrives at $V_\textrm{reset}$ = $-$2.2 V, the Au/LNFO/ITO/glass device switches from LRS to HRS. When the negative bias voltage sweeps back, the relationships of *I* and *V* are similar to that in the low positive region, namely, $I \propto V^{n}$ ($n=\sim$11.78), $I \propto V^{2}$ and $I \propto V$, respectively. Figure 4 shows the conversion between Ohmic law ($I \propto V$) and Child’ law ($I \propto V^{2}$), indicating that traps existent in the LNFO layer. With different operating voltages, the filling rate of the traps is different due to their different energy levels in the LNFO film. It leads to the multilevel RS performance in the Au/LNFO/ITO/glass device [30].

**Fig. 5:**
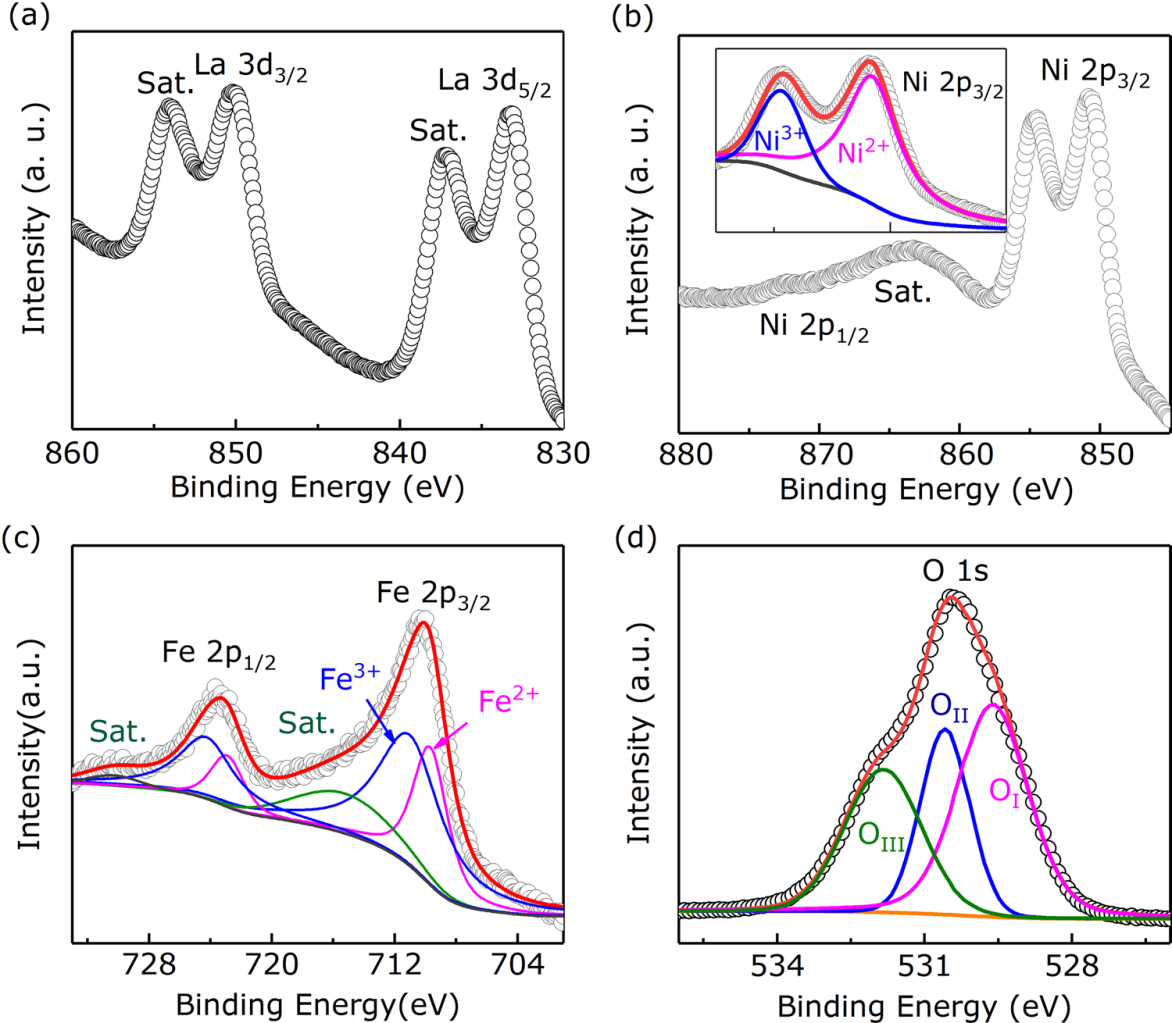
X-ray photoelectron spectra of **a** La 3*d*, **b** Ni 2*p*, **c** Fe 2*p* and **d** O 1 *s* for LNFO films

In order to verify the type of traps in the LNFO-based device, XPS was characterized to investigate the chemical valence states of La, Ni, Fe and O in the LNFO layer. Figure 5a–d present La, Ni, Fe and O core-level XPS spectra, respectively. Figure 5a exhibits two spin-orbit peaks two spin-orbit peaks of La 3*d*, corresponding to 3$d_\mathrm{5/2}$ and 3$d_\mathrm{3/2}$, with two shake-up satellites (denoted as Sat.). The peaks located at the binding energy of 833.74 eV (3$d_\mathrm{5/2}$) and 850.78 eV (3$d_\mathrm{3/2}$) are corresponding to La$^{3+}$, respectively [12]. The satellite peaks appearing with the binding energy of 837.81 eV for 3$d_\mathrm{5/2}$ and 854.69 eV for 3$d_\mathrm{3/2}$ mainly are resulting from the electrons transfer from O 2*p* to La 4*f* [12, 37]. Figure 5b shows the characteristic peaks of Ni 2$p_\mathrm{3/2}$ appear in the range of 858.0$\sim$845.0 eV, and the peak of Ni 2$p_\mathrm{1/2}$ appears at the BE of 872.5 eV. The fitting results of the Ni 2$p_\mathrm{3/2}$ XPS spectrum shown in the inset of Fig. 5a clearly exhibit that the valence state of the Ni ions is not normally considered to be Ni$^{3+}$ (850.67 eV) in the LNFO film but there is a coexistence of Ni$^{3+}$ and Ni$^{2+}$. The Ni$^{3+}$ peak position locates at 854.65 eV and the Ni$^{2+}$ peak is at 850.78 eV (2$p_\mathrm{3/2}$). As shown in Fig. 5c, the fitting peaks of Fe 2*p* spectrum clearly exhibits that the valence state is not normally considered to be Fe$^{3+}$ but there is a coexistence of Fe$^{3+}$ and Fe$^{2+}$, Fe$^{3+}$ peak-positions are at 727.65 eV (2$p_\mathrm{3/2}$) and 713.90 eV (2$p_\mathrm{1/2}$), Fe$^{2+}$ peaks locate at 722.56 eV (2$p_\mathrm{3/2}$) and 709.50 eV (2$p_\mathrm{1/2}$). The origin of mix valences coexistence in Fe and Ni elements is usually charge compensation of OVs, which is common in perovskite oxides [38, 39]. Figure 5d illustrates the fitting XPS spectrum of O 1*s*. the asymmetric O 1*s* peak can be fitted with three peaks centered at 529.61 eV, 530.58 eV and 531.83 eV, which are name O$_\textrm{I}$, O$_\textrm{II}$ and O$_\textrm{III}$, respectively. As previous researches, the peak O$_\textrm{I}$ located at 529.61 eV is lattice oxygen, the peak O$_\textrm{II}$ centered at 530.58 eV can be assigned to VO [40]. The less-intense peak O$_\textrm{III}$ can be supposed to adsorbed oxygen [40]. OVs can play important roles in the oxides layer, such as trap centers, carriers *etc*., to participate in the RS processes of oxides-based RS devices [41–43]. Thus, we speculated that the traps involved in conductive processes of the LNFO-based RS device were OVs.

**Fig. 6:**
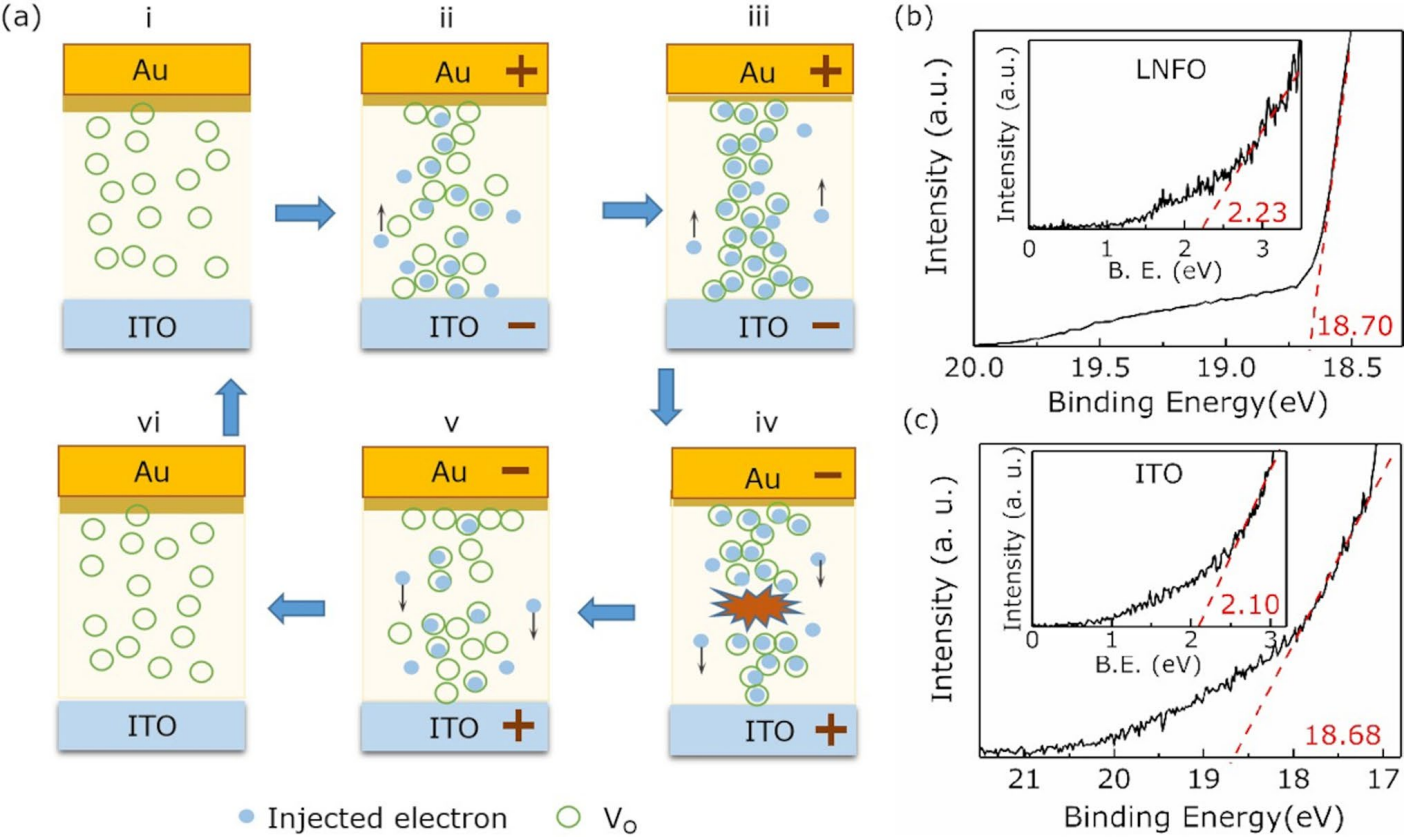
**a** A schematic diagram of the conductive process in the Au/LNFO/ITO heterojunction under bias voltage sweeps. (i) initial state corresponding to HRS; (ii)–(iii) SET process; (iv)–(vi) electrical RESET process. The UPS spectra of the LNFO film (**b**) and ITO substrate **c** at the cut-off region obtained with the He I source ($h\nu$ = 21.22 eV). The insets present Fermi edges of LNFO and ITO, respectively

Initially, OVs disperse randomly in the LNFO layer, the Au/LNFO/ITO/glass device keeps HRS, as shown in Fig. 6(a) i. At a low sweep voltage region (0$\sim$0.3 V), the conductive behavior is dominated by the thermally generated free carriers in the LNFO layer, and the relationship of *I–V* follows Ohm’s law, $I\propto V$. With the sweep bias voltage increasing (0.3 V$\sim$0.7 V), more electrons can be injected from the ITO electrode due to the weak contact barrier. And then, the concentration of injected electrons is higher than that of intrinsic carriers in the LNFO layer. OVs traps capture injected electrons and migrate toward ITO electrode gradually to form conductive channels in the LNFO layer, as shown in Fig. 6(a) ii. The LNFO layer is in the trap-half-filled state in the bias voltage region (0.3 V$\sim$0.7 V), and the relationship of *I–V* follows Child’s law, $I\propto$ $V^{2}$ [35, 44]. In this conductive process, the current behavior obeys the shallow-trap-controlled SCL conduction [34]. With the bias voltage increasing further (0.8 V$\sim$2 V), the additional injected electrons can fill the empty traps and jump out immediately due to their high activation energy; OVs trap centers are considered as filling up, as shown in Fig. 6(a) iii. The slope of *I–V* increases sharply ($\textit{I}\propto \textit{V}^{n}$). In this conductive process, the current behavior obeys the trap-free SCL conduction, which is controlled by the traps dispersed exponentially in band gap [34, 44]. Above conductive processes, where the relationship of *I–V* are $\textit{I}\propto \textit{V}^{2}$ and $\textit{I}\propto \textit{V}^{n}$, are classified into the trapping process [30, 34]. When the bias voltage crosses $V_\textrm{set}=2.2 V$, the Au/LNFO/ITO/glass device switches HRS to LRS. The device keeps LRS due to the high concentration of electrons in the LNFO layer, even though the voltage sweeps reversely. When the voltage crosses $V_\textrm{reset}=-2.2 V$ and decreases further, the trapped electrons will be discharged from OVs and the channels formed by OVs are fused, as shown in Fig. 6(a) iv. The device switches from LRS to HRS. And then, the relationships of *I–V* recovers $I\propto$ $V^{n}$ and $I\propto$ $V^{2}$, respectively. Above conductive process is named the detrapping process [30, 34] With the sweeping voltage decreasing further, the concentration of injected electrons decreases continually, which is lower than that of thermally generated electrons. And then, the relationship of *I–V* recovers the Ohm’s law, as shown in Fig. 6(a) v and vi.

Moreover, the asymmetry of the *I–V* curves in Au/LNFO/ITO/glass devices can be assigned to the modulation of Schottky-like contact. As shown in Fig. 6b, the work function of LNFO is calculated to be 4.75 eV that is closed to the calculated value of ITO (4.64 eV) from Fig. 6c. Thus, the contact between LNFO and ITO is quasi-Ohmic; the contact ofAu/LNFO is Schottky-like due to the higher work function of ($\sim 5.40$ eV) [45]. Under a positive voltage, electrons drift to the Au/LNFO interface and can be captured by OVs in the dissipative region near this interface. It results in the reducing of Schottky-like barrier. With the positive voltage further increasing, the barrier reduces further, resulting the tunneling current is enlarged and an quasi-Ohmic contact is formed in the Au/LNFO interface. And then, electrons inject from Au to LNFO easily; the Au/LNFO/ITO/glass device switches from HRS to LRS. When a negative voltage is adopted to Au electrode sufficiently, the electrons can be released from OVs located at the dissipative region and more OVs migrate to Au/LNFO interface, resulting the Schotty-like barrier is recovered; the concentration of injected electrons decreases in the LNFO layer due to the hindrance of barrier. When the negative voltage reaches $V_\textrm{reset}$, the device switches from LRS to HRS.

## Conclusion

Dense and flat LNFO films were fabricated on the ITO/glass substrate by sol–gel method. The BRS behavior could be maintain in 100 cycles and remained after 30 days, indicating that the LNFO-based RS device owned good memory stability. Surprisingly, multilevel RS characteristics were firstly observed in the Au/LNFO/ITO/glass device. The HRSs and LRS with the maximum ratio of $\sim$500 could be maintained stably over 900 s and 130 cycles, indicating the fine retention and endurance ability of multilevel switching in this LNFO-based RS device. The BRS behavior of Au/LNFO/ITO/glass devices obeyed the SCLC model controlled by OVs dispersed in the LNFO layer. With operating voltages, injected electrons could be captured or released by OVs in the LNFO layer during the trapping or detrapping process. Thus, the resistance state of the Au/LNFO/ITO/glass device switched between HRS and LRS reversibly. In addition, the change in Schottky-like barrier generated at Au/LNFO interface could also modulate the switchover of resistance states. The multilevel RS performance of Au/LNFO/ITO/glass devices can provide an opportunity for researching deeply on the high density RS memory in lead-free double perovskite oxides-based devices.

### Supplementary Information

Below is the link to the electronic supplementary material.

Supplementary file 1 (DOCX 109 KB)

## Data Availability

All data generated and analyzed during this study are included in this article.
