# Supplementary material for: Multilevel resistive switching memory in lead-free double perovskite La2NiFeO6 films
Source: Discov Nano. 2023 Aug 29;18(1):107. doi: 10.1186/s11671-023-03885-7 (PMC10465475; doi:10.1186/s11671-023-03885-7)
Supplement: Supplementary file 1 — (DOCX 109 KB) [file 11671_2023_3885_MOESM1_ESM.pdf]

Multilevel resistive switching memory in lead-free double perovskite La_2NiFeO_6 films

Yongfu Qin, Yuan Gao†, Fengzhen Lv, Fangfang Huang, Fuchi Liu, Tingting Zhong, Yuhang Cui, Xuedong Tian

College of Physical Science and Technology and Guangxi Key Laboratory of Nuclear Physics and Technology, Guangxi Normal University, Yucai Road, Guilin, 541000, China.

*Corresponding author(s).

Email(s): lvfzh17@mailbox.gxnu.edu.cn; liufuchi@gxnu.edu.cn; snowtxd@gxnu.edu.cn;

† This author contributed equally to this work.

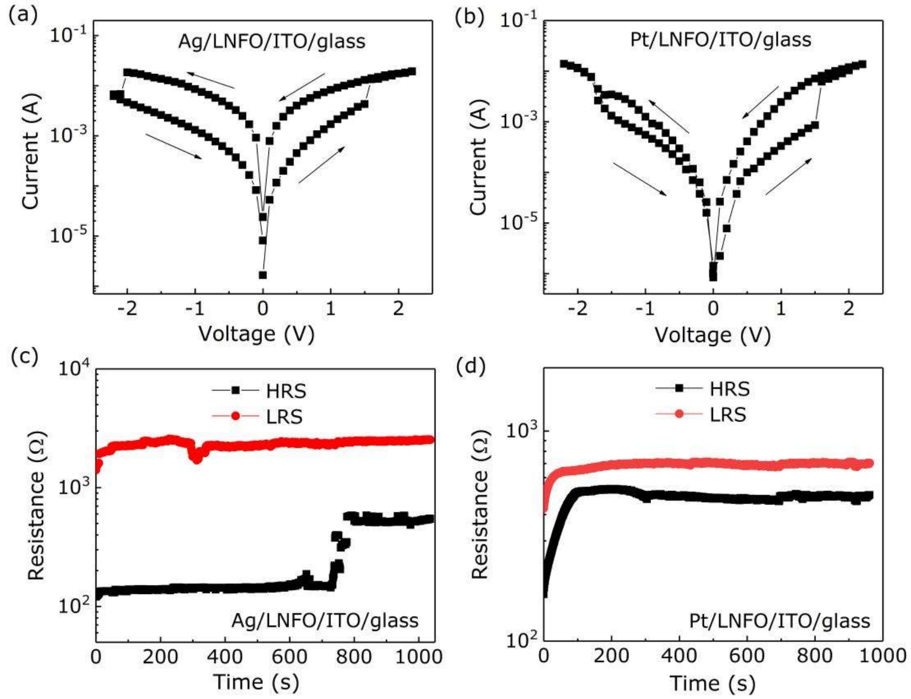

Fig. S1 *I-V* curves of LNFO-based memory device with (a) Ag electrode and (b) Pt electrode. Resistance *vs.* retention time in (c) Ag/LNFO/ITO/glass and (d) Pt/LNFO/ITO/glass.
